# Factors associated with quality of life in patients with Chagas disease: SaMi-Trop project

**DOI:** 10.1371/journal.pntd.0008144

**Published:** 2020-05-27

**Authors:** Nayara Dornela Quintino, Ester Cerdeira Sabino, José Luiz Padilha da Silva, Antonio Luiz Pinho Ribeiro, Ariela Mota Ferreira, Gabriela Lemes Davi, Claudia Di Lorenzo Oliveira, Clareci Silva Cardoso

**Affiliations:** 1 School of Medicine, Federal University of São João del-Rei, Divinópolis, Minas Gerais, Brazil; 2 Institute of Tropical Medicine, University of São Paulo, São Paulo, Brazil; 3 Department of Statistics, Federal University of Paraná, Curitiba, Paraná, Brazil; 4 Clinical Hospital and School of Medicine, Federal University of Minas Gerais, Belo Horizonte, Brazil; 5 Graduate Program in Health Sciences, State University of Montes Claros, Montes Claros, Minas Gerais, Brazil; Instituto de Ciências Biológicas, Universidade Federal de Minas Gerais, BRAZIL

## Abstract

Chagas disease (CD) is a neglected tropical disease and its main consequence is chronic chagasic cardiomyopathy (CCC). The clinical manifestations of CD can cause functional limitation and impact on the perception of quality of life (QoL). We evaluated the QoL profile of patients with CD living in remote regions, and their association with socio-demographic, behavioral, and clinical characteristics.A cross-sectional study, using the baseline of a cohort study (SaMi-Trop) was conducted in the state of Minas Gerais, Brazil, an endemic region for CD. The cohort is composed of 1959 patients with CD. The patients were interviewed, and blood collection and electrocardiogram (ECG) were performed. The WHOQOL-BREF scale was used to assess QoL. Descriptive statistics were performed and, for the QoL scale, measures of central tendency and dispersion were calculated. Inflated beta regression models constructed using the gamlss framework were used, and fitted using R software. A significance level of 5% for all analyzes was adopted.QoL was evaluated in 625 participants. The Environment (57.66) and Social Relationships (73.17) domains presented the lowest and highest QoL score respectively. There was no statistically significant difference between QoL domains in the presence of CCC. In the multivariate analysis, the factors associated with a lower QoL were in the Physical domain age increase (OR: 0.95, CI: 0.91–0.99) and using an angiotensin converting enzyme inhibitor (OR 0.89; CI: 0.80–0.99); in the Social Relations domain a history of acute myocardial infarction (OR: 0.75; CI: 0.61–0.92); and in the Environment domain age increase (OR 0.94, CI 0.91–0.97). The factor associated with higher QoL was the use of angiotensin receptor blockers (OR: 1.15; CI 1.04–1.26).The findings of this study reinforce the importance of QoL indicators for planning care and clinical management of patients from remote regions with CD.

**Trial registration:**

NCT02646943.

## Author summary

Chagas disease (ChD) is a neglected tropical disease, with the majority of the individuals affected living in Latin America. Functional limitation and poor perception of quality of life (QoL) may be identified. QoL indicators are important for planning and to improve patients care. However there is a gap in the literature regarding the evaluation of QoL in patients living with chronic CD, in remote regions assisted by primary health care in their territory of residence. A cross-sectional study was conducted using the baseline of a prospective cohort performed in remote towns in Minas Gerais state, Brazil. Lower QoL scores were identified in the Environment domain followed by the Physical domain. Higher scores were found in the Social Relations domain. Low QoL scores have been associated with clinical aspects such as use of angiotensin converting enzyme inhibitor (ACEI), and history of acute myocardial infarction, and socio-demographic aspects such as increasing age. QoL is positively associated with medicine use of angiotensin receptor antagonists (ARA). The subjective perception of QoL was not associated with the severity of the disease using as a marker the presence of CCC in the evaluated population.

## Introduction

Chagas disease (CD) is a neglected tropical disease with approximately 6 to 7 million affected individuals worldwide, most in Latin America [1,2]. It is an infectious condition with acute and chronic phase. The most important consequence in the chronic phase is chagasic cardiomyopathy (CCC) occurring in 20–30% of infected persons [3] with an incidence rate of 1.85% person-years [4]. Severe manifestations, including arrhythmia, heart failure, thromboembolism, and sudden death [1,5,6] may occur in CCC. These manifestations are associated with increased myocardial impairment and can cause functional limitations with social, economic, and psychological impacts, among them, on the individual perception of quality of life (QoL) [7–9].

Evaluating QoL in patients with CD is extremely important considering the social vulnerability of this population, which is presented with unfavorable socio-demographic, economic, and living conditions. Moreover, it is common for these patients to live in remote regions with little access to specialized health services [10–12]. The improvement of QoL is one of the therapeutic objectives and its indicators are relevant to guide decisions and behaviors of health teams in order to improve patient care, contributing not only to a better diagnosis and clinical management, but also to the planning of public policies and the allocation of resources [13,14].

Although the production of QoL indicators in chronic disease has been shown to be relevant, there is a gap in the literature regarding the evaluation of QoL in patients living with chronic CD, in remote regions with difficulty accessing specialized health services attended by primary health care (PHC) in their territory of residence. Most QoL studies with this population are performed in specialized services [7–9] which is a limitation for the generalization of the findings considering that this can overestimate the perception of QoL. Therefore, the objective of this study is to evaluate the QoL profile of patients with CD living in remote regions, and its association with socio-demographic, behavioral, and clinical profile.

## Methods

### Ethics statement

This research was approved by the Ethics Committee in Research, number 179.685/2012 (National Commission of Ethics in Research, CONEP). In this investigation, all human subjects were adults who had given written informed consent.

### Study design

This is a cross-sectional study using the baseline (2013–2014) of a prospective cohort study called the SaMi-Trop project (*Centro de Pesquisa em Medicina Tropical São Paulo/Minas Gerais*) [15]. This cohort is a multicenter study resulting from the cooperation between four Brazilian public universities (USP, UFMG, UFSJ, and UNIMONTES).

### Study population and recruitment

The cohort was established in an endemic area for CD in the state of Minas Gerais, Brazil. It was composed of patients from 21 municipalities with high prevalence of the disease, located in remote regions, with low socioeconomic and health indicators. Most of the municipalities are small, with high coverage from the Primary Health Care Program [16]. Health care in Brazil is organized into regionalized Health Care Networks [17], with primary care services available in all municipalities. Specialized health services are regionalized and organized in larger cities that serve as a reference for the region.

The SaMi-Trop cohort is composed of patients assisted by the Telehealth Network of Minas Gerais (*Rede de Telessaúde de Minas Gerais*—RTMG), a program to support primary health care (PHC) in Brazil [18]. This program offers tele-electrocardiogram activities where all electrocardiographic information from patients is sent to a central reading unit that also collects clinical data such as history of CD. Using this database for the period from 2011 to 2012, we selected the 21 municipalities with a high prevalence of self-reported CD. A total of 4689 patients were eligible for the cohort, and 2157 were located and completed the initial evaluation in 2013–2014. The sample size estimated to observe the main outcomes in this cohort was 2,000 individuals [15].

All baseline participants were evaluated for the presence of anti-*T*.*cruzi* antibodies using chemiluminescent microparticle immunoassay. Negative results were reassessed, and immuno-negative results were confirmed by two additional chemiluminescent immunoassays using different antigens. The eligibility criteria for QoL interview were individuals able to understand the QoL questions and conduct the self-assessment of their QoL.

### Data collection and variables

The baseline was collected between June 2013 and July 2014. Patients were recruited by professionals from the Family Health Program, who scheduled the day and time for the interview and clinical evaluation. The interviews were conducted by previously trained health professionals.

The data collected included: a) socio-demographic and clinical information, and health related behaviors (tobacco use, alcohol consumption, and physical activity); b) indicators of quality of life measured by the WHOQOL-Bref scale [19]; c) electrocardiographic information; and d) blood collection (immunoassays, polymerase chain reaction (PCR) for *T*. *cruzi* and N-terminal prohormone of natriuretic peptide type B (NT-ProBNP)).

In this study, the primary outcome was quality of life through its four domains (Physical, Psychological, Social Relations and Environment). The explanatory variables were socio-demographic (gender, age, skin color, schooling, marital status, monthly family income, number of people at home); behavioral (physical activity, alcohol consumption, tobacco use); and clinical (time since CD diagnosis, electrocardiogram (ECG) changes typical for CD, presence of CCC and comorbidities, pacemaker use, number of medications and class, benznidazole use, health self-perception, NT-ProBNP levels, ECG changes typical for CD + NT-ProBNP).

ECG changes typical for CD were classified using the Minnesota Code (MC) criteria [20]. All ECG measurements and codes were manually reviewed by a trained cardiologist. ECG changes considered to be typical for CCC include [21]: complete intraventricular block (7.1, 7.2, 7.4 or 7.8); frequent ventricular premature beats (MC 8.1.2 or 8.1.3); atrial fibrillation or flutter or supraventricular tachycardia (MC 8.3.x. or 8.4.2); other major arrhythmias (MC 8.2.x, except 8.2.1); major atrioventricular conduction abnormalities or pacemaker use (MC 6.1, 6.2.x, 6.4, 6.8, 8.6.1 or 8.6.2); major Q wave abnormalities [MC 1.1.x or 1.2.x] or minor Q wave abnormalities with ST segment or T-wave abnormalities [1.3.x and 4.1.x, 4.2, 5.1 or 5.2]; or major isolated ST segment or T-wave abnormalities (MC 4.1.x, 4.2, 5.1 or 5.2). The level of NT-ProBNP (Roche diagnosis) was categorized according to age specific cutoff points for heart failure (HF) [22,23]. CCC classification was performed according to the following parameters: 1) non-chagasic cardiomyopathy (ECG with non-typical change for CD); 2) CCC without HF (ECG with changes typical for normal CD and NT-ProBNP); and 3) CCC with HF (ECG with changes typical for CD and high NT-ProBNP).

QoL was assessed by the WHOQOL-Bref scale, a validated instrument for Brazil that has good psychometric qualities, such as validity and reliability [19]. It consists of 26 questions, being 2 generic questions (QoL self-assessment and satisfaction with health), and 24 questions distributed in four areas: Physical, Psychological, Social Relations and Environment. The Physical domain (7 questions) assesses aspects related to physical capacity such as: pain, fatigue, sleep, mobility and activities of daily living, ability to work, and use of medications. The Psychological domain (6 questions) assesses aspects such as memory, concentration, body self-perception, and positive and negative feelings. The Social Relations domain (3 questions) evaluates social relations, social support and sexual activity. The Environment domain (8 questions) involves the perception of physical security and protection, home environment, financial resources, health and social care, and leisure.

QoL is assessed on a Likert scale in five points. The higher the score, the better the individual’s perception of QoL in the previous 15 days. Considering the low educational level of the patients, the scale was applied by an interviewer. This strategy is also used in research with neglected disease [24].

### Statistical analysis

The results of the QoL evaluation in each domain were transformed into a linear scale from 0 to 100. The explanatory variables were grouped into socio-demographic, behavioral, and clinical characteristics. Frequency distribution, measures of central tendency, and dispersion were calculated. Inflated beta regression models belonging to the Generalized Additive Models for Location Scale and Shape (GAMLSS) [25] were constructed for comparison of the QoL domains and classification of cardiomyopathy, and later to evaluate the relationship between each response and covariates. Wald type tests and Likelihood Ratio tests were considered. The logit link function was adopted, in which the exponential of the parameters is interpreted in terms of odds ratio (OR). An OR < 1 indicates lower QoL and an OR > 1 represents better QoL.

The explanatory variables with p value ≤ 0.20 and variables with clinical relevance (gender, time since diagnosis of CD, ECG typical for CD, NT-ProBNP) were included in the model. Collinearity was tested between the variables and those highly correlated were excluded. The covariates with p value <0.05 remained in the final model. The model's goodness-of-fit was evaluated by means of plots of quantile residuals, Q-Q plots, and summary statistics.

In order to evaluate differential bias, the individuals who assessed the QoL were compared with those who did not considering socio-demographic, behavioral, and clinical characteristics. The groups were compared using Mann-Whitney and chi-squared tests, as appropriate.

All analyses were conducted with the free R software version 3.4.3, using the foreign and plyr packages (data importing and manipulation), ggplot2 and gridExtra (graphs), and gamlss (model fitting).

## Results

### Study patients

Of the 1959 seropositive individuals in the SaMi-Trop cohort, 1334 were not included because they were unable to assess their QoL due to difficulties in understanding the instrument (cognitive and language difficulties). Thus, a total of 625 patients completed the interview, representing less than one third of the SaMi-Trop cohort patients. The socio-demographic, behavioral, and clinical characteristics of the patients are described in Table 1. Those who answered the QoL evaluation interview were compared to those who did not for the clinical and socio-demographic variables. Those who participated in the QoL assessment presented higher age, were literate, and more time since CD diagnosis. Although longer diagnosis of CD and use of amiodarone was observed in individuals who had the QoL assessed, no statistically significant differences were found in clinical outcomes.

**Table 1:** Socio-demographic, behavioral, and clinical characteristics of the individuals included (n = 625) and not included (n = 1334) in the QoL evaluation.

Variables	Individuals included	Individuals not included	p-value
	N %	N %	
**Socio-demographic**			
Mean age (n = 625)	56.7 (±12.2)*	59.8 (±12.8)	< 0.001
Mean monthly family income (n = 618)	437.10 (±225.24)*	407.62 (±206.11)	0.118
Mean number of people in the home (n = 625)	3.5 (±1.7)*	3.6 (±1.9)*	0.859
**Gender**			
Female	411/625 (65.8)	912/1334 (68.4)	0.273
**Skin color**			
Mixed	386/625 (61.8)	758/1328 (57.0)	0.011
White	141/625 (22.6)	285/1328 (21.5)	
Others^a^	98/625 (15.7)	285/1328 (21.5)	
**Literate**			
Yes	405/625 (64.8)	687/1327 (51.8)	< 0.001
**Marital Status**			
Married/living with partner	398/625 (63.7)	840/1328 (63.3)	0.360
Widowed	134/625 (21.4)	315/1328 (23.7)	
Single and divorced	93/625 (14.9)	173/1328 (13.0)	
**Behavioral**			
**Practice Physical Activity**^**b**^			
Yes	155/624 (24.8)	279/1322 (21.1)	0.074
**Alcohol Use**^**c**^			
Yes	107/619 (17.3)	178/1151 (15.5)	0.354
**Tobacco Use**			
Never smoked	372/624 (59.6)	931/1324 (70.3)	< 0.001
Ex-smoker	195/624 (31.3)	307/1324 (23.2)	
Smoker	57/624 (9.1)	86/1324 (6.5)	
**Clinical**			
**Time since CD diagnosis** (years)			
> 5	520/613 (84.8)	1036/1283 (80.7)	0.036
**ECG typical for CD**			
Yes	356/607 (58.6)	743/1303 (57.0)	0.535
**Cardiomyopathy classification**			
CC non chagasic	251/604 (41.6)	560/1302 (43.0)	0.691
CCC without HF	290/604 (48.0)	598/1302 (45.9)	
CCC with HF	63/604 (10.4)	144/1302 (11.1)	
**Comorbidities** (Yes)			
Hypertension	408/625 (65.3)	845/1334 (63.3)	0.434
Diabetes mellitus	66/625 (10.6)	132/1334 (9.9)	0.708
History of AMI	31/625 (5.0)	61/1334 (4.6)	0.793
**Use of Pacemaker**			
Yes	38/619 (6.1)	82/1305 (6.3)	0.983
**Medications in use** (n)			
1–2	239/625 (38.2)	475/1334 (35.6)	0.288
3–4	154/625 (24.6)	384/1334 (28.8)	
5 or more	38/625 (6.1)	80/1334 (6.0)	
None	194/625 (31.0)	395/1334 (29.6)	
**Medication class** (Yes)			
Diuretic	296/620 (47.7)	655/1315 (49.8)	0.424
ACEI	188/620 (30.3)	365/1315 (27.8)	0.266
Amiodarone	177/625 (28.3)	252/1325 (19.0)	< 0.001
ARA	157/620 (25.3)	393/1316 (29.9)	0.044
Digoxin	46/621 (7.4)	94/1319 (7.1)	0.897
Beta blocker	45/619 (7.3)	95/1317 (7.2)	1.000
**Use of benznidazole**			
Yes	157/591 (26.6)	335/1220 (25.2)	0.757
**Self perception of health**			
Bad/Very bad	414/624 (66.3)	909/1322 (68.8)	0.091
Very good	163/624 (26.1)	336/1322 (25.4)	
Good/Average	47/624 (7.5)	67/1322 (5.1)	
**High levels of NT-ProBNP**			
Yes	67/624 (10.7)	162/1331 (12.2)	0.399
**ECG typical for CD + high levels of NT-ProBNP**			
Yes	63/604 (10.4)	144/1300 (11.1)	0.715

* standard deviation (SD)

^a^ Others: black (14.1%), oriental (1.4)

^b^ Practicing physical activity refers to the practice of exercise or any physical activity, such as sport (for example: football, tennis, running, swimming, etc.)

^c^ alcohol use in the last month

CD = Chagas Disease, CCC = Chronic Chagasic Cardiomyopathy, CC = Chronic Cardiomyopathy, ECG = Electrocardiogram, AMI = Acute Myocardial Infarction, ACEI = angiotensin converting enzyme inhibitor, ARA = angiotensin receptor antagonists, NT-ProBNP = N—B-type natriuretic peptide prohormone terminal.

Dollar quotation for July 2013 (US$ 1.00 = R$ 2.252)

Of the 625 patients who answered the QoL evaluation, 65.8% were females with a mean age of 56.7 (± 12.2), income of U$ 437.10 (± U$ 225.24), being mostly married or living with a partner (63.7%). Of these 625 patients, 59.6% never smoked and 31.3% were ex-smokers. Regarding the clinical characteristics, 84.8% reported having more than 5 years of diagnosis of CD, and 58.6% of the patients presented changes typical for ECG compatible with CD. The hypertension comorbidity was the most prevalent (65.3%) and 38.2% of the patients used 1 to 2 medications, with diuretics being the most used class (47.7%), followed by the angiotensin converting enzyme inhibitor (ACEI) (30.3%), amiodarone (28.3%), and angiotensin receptor antagonists (ARA) (25.3%). The use of the anti-parasitic medication benznidazole was reported by 26.6% of the patients. In 10.7% of the patients NT-ProBNP was observed with alterations suggestive of heart failure and 10.4% of patients presented a combined ECG outcome typical for CD and elevated levels of NT-ProBNP.

### Primary and secondary outcomes

The distribution of QoL scores by domains and by cardiomyopathy classification is shown in Table 2. The QoL scores in the four domains ranged from 57.66 (Environment domain) to 73.17 (Social Relations). However, there was no statistically significant difference in the subjective perception of QoL when the presence of chagasic and non-chagasic cardiomyopathy was compared (p> 0.05).

**Table 2:** Total Quality of Life Distribution by WHOQOL-BREF domain, according to the classification of cardiomyopathy.

QoL Domains		Classification of cardiomyopathy (n = 604)			p-value*
	Total (n = 625)	CC non chagasic	CCC without HF	CCC with HF	
	Mean (DP)	Mean (DP)	Mean (DP)	Mean (DP)	
Physical	57.84 (15.32)	58.54 (15.11)	56.81 (15.15)	60.54 (15.17)	0.177
Psychological	65.98 (12.85)	65.41 (13.08)	66.65 (12.31)	66.20 (13.76)	0.304
Social Relations	73.17 (13.99)	73.57 (14.59)	72.39 (13.75)	73.15 (13.42)	0.819
Environment	57.66 (12.26)	57.33 (12.66)	57.58 (11.89)	58.13 (12.29)	0.959

Note:

* p-value refers to the comparison of QoL domains and CCC classification

In the bivariate analysis (Tables 3 and 4), the variables selected for inclusion in the multivariate analysis (p<0.20) were: 1-Physical domain: age, marital status, number of medications, use of ACEI, and combined ECG endpoint for CD + NT-ProBNP; 2- Psychological domain: marital status, history of smoking, changes typical for CD in ECG, number of medications, and use of ACEI; 3- Social Relations domain: gender, schooling, history of alcohol use, history of AMI, and use of ARA; 4- Environment domain: age, skin color, schooling, history of alcohol use, history of AMI, use of ACEI, and self-perception of health.

**Table 3:** Bivariate analysis using inflated beta regression by WHOQOL-BREF domain, considering the socio-demographic and behavioral characteristics of patients with Chagas disease (n = 625).

Variables	WHOQOL-BREF Domains			
	Physical	Psychological	Social Relations	Environment
	OR (IC 95%)	OR (IC95%)	OR (IC95%)	OR (IC95%)
**Socio-demographic**				
Age^a^ (n = 625)	0.96 (0.92–0.99)*	1.00 (0.96–1.04)	1.00 (0.96–1.04)	0.95 (0.92–0.99)*
Income (n = 618)	1.00 (1.0–1.0)	1.00 (1.00–1.00)	1.00 (1.00–1.00)	1.00 (1.00–1.00)
No. of people in the home	1.01 (0.98–1.04)	0.99 (0.97–1.02)	0.98 (0.95–1.01)	1.00 (0.98–1.02)
**Gender**				
Female	1.02 (0.92–1.13)	1.04 (0.95–1.14)	1.10 (1.00–1.22)*	1.03 (0.94–1.12)
**Skin color**				
White	(ref)			
Mixed	1.00 (0.89–1.13)	1.03 (0.92–1.14)	1.04 (0.92–1.17)	1.05 (0.95–1.16)
Others^b^	1.02 (0.87–1.20)	1.01 (0.88–1.17)	0.97 (0.83–1.13)	1.09 (0.96–1.25)**
**Literate**				
Yes	1.04 (0.94–1.15)	1.05 (0.96–1.16)	1.09 (0.98–1.20)**	1.06 (0.97–1.15)**
**Marital Status**				
Married	(ref)			
Widowed	0.81 (0.72–0.91)*	0.96 (0.86–1.07)	0.96 (0.85–1.08)	0.93 (0.84–1.03)**
Single and divorced	0.91 (0.79–1.05)	1.09 (0.96–1.24)**	0.98 (0.85–1.12)	1.02 (0.90–1.14)
**Behavioral**				
**Physical activity**				
Yes	0.96 (0.86–1.08)	0.99 (0.89–1.10)	1.00 (0.90–1.12)	0.98 (0.91–1.08)
**Alcohol Use**^**c**^				
Yes	1.03 (0.90–1.17)	1.03 (0.92–1.16)	1.10 (0.97–1.25)**	1.09 (0.97–1.21)**
**Smoking**				
Never smoked	(ref)			
Ex smoker	1.01 (0.90–1.12)	1.09 (0.98–1.20)**	1.02 (0.92–1.13)	0.99 (0.91–1.08)
Smoker	1.08 (0.91–1.29)	0.99 (0.84–1.17)	0.99 (0.84–1.17)	0.97 (0.84–1.12)

* p <0.05

** p <0.20

^a^ age (in 10 years)

^b^ other: black and oriental skin color

^c^ alcohol use in the last month

**Table 4:** Results of bivariate analysis using inflated beta regression by WHOQOL-BREF domain according to the clinical characteristics of patients with Chagas disease (n = 625).

Variables	WHOQOL-BREF Domains			
	Physical	Psychological	Social Relations	Environment
	OR (IC95%)	OR (IC95%)	OR (IC95%)	OR (IC95%)
**Time since CD diagnosis** (years)				
≤ 5	(ref)			
> 5	0.93 (0.81–1.07)	1.02 (0.90–1.16)	1.02 (0.89–1.17)	0.94 (0.84–1.05)
**ECG typical for CD**				
Yes	0.96 (0.86–1.06)	1.07 (0.98–1.17)**	0.97 (0.88–1.07)	1.01 (0.93–1.10)
**Classification of cardiomyopathy**				
CC non chagasic (ref)				
CCC without HF	0.93 (0.84–1.04)	1.07 (0.98–1.18)	0.97 (0.88–1.07)	1.01 (0.93–1.10)
CCC with HF	1.11(0.94–1.31)**	1.03 (0.89–1.20)	1.01(0.87–1.19)	1.01 (0.88–1.15)
**Comorbidities**(Yes)				
Hypertension	0.95 (0.86–1.05)	0.94 (0.86–1.03)	1.02 (0.92–1.12)	0.95 (0.88–1.04)
Diabetes mellitus	0.95 (0.81–1.12)	1.00 (0.86–1.15)	0.94 (0.81–1.10)	0.95 (0.84–1.09)
History of AMI	0.96 (0.76–1.20)	0.99 (0.81–1.22)	0.74 (0.60–0.91)*	0.87 (0.72–1.04)**
**Pacemaker use**				
Yes	1.03 (0.84–1.26)	1.03 (0.86–1.25)	0.92 (0.76–1.12)	0.92 (0.78–1.09)
**Medications in use (n)**				
None	(ref)			
1–2	0.96 (0.86–1.09)	1.00 (0.89–1.11)	1.02 (0.91–1.14)	0.96 (0.87–1.06)
3–4	0.90 (0.79–1.03)**	0.91(0.81–1.03)**	0.95 (0.84–1.08)	0.98 (0.88–1.09)
5 or more	0.94 (0.75–1.16)	1.09 (0.89–1.33)	0.91 (0.74–1.12)	1.03 (0.86–1.23)
**Class of medication (Yes)**				
Diuretic	1.00 (0.91–1.11)	1.00 (0.91–1.09)	0.98 (0.89–1.07)	1.01 (0.93–1.10)
Digoxin	0.95 (0.79–1.14)	1.01 (0.85–1.20)	1.01 (0.84–1.21)	0.98 (0.84–1.14)
Amiodarone	1.05 (0.94–1.17)	0.97 (0.88–1.07)	0.99 (0.90–1.10)	1.02 (0.93–1.12)
Beta blocker	0.94 (0.78–1.14)	1.00 (0.84–1.19)	0.99 (0.82–1.18)	0.93 (0.80–1.09)
ACEI	0.88 (0.79–0.98)*	0.93 (0.85–1.03)**	0.96 (0.87–1.06)	0.92 (0.84–1.01)**
ARA	1.04 (0.93–1.16)	1.04 (0.94–1.16)	1.08 (0.97–1.21)**	1.12 (1.02–1.23)*
**Benznidazole use**				
Yes	1.10 (0.98–1.23)	1.04 (0.94–1.16)	0.97 (0.87–1.08)	1.01 (0.92–1.11)
**Self-perception of health**				
Good/average	(ref)			
Bad/very bad	1.01 (0.84–1.22)	1.07(0.90–1.26)	0.93 (0.78–1.12)	1.15 (0.98–1.34) **
Very good	0.91(0.74–1.11)	1.09(0.91–1.31)	0.94 (0.77–1.15)	1.06 (0.90–1.25)
**High levels of NT-ProBNP**				
Yes	1.07 (0.92–1.26)	1.00(0.87–1.16)	0.97 (0.83–1.13)	1.00 (0.88–1.14)
**ECG typical for CD + high levels of NT-ProBNP**				
Yes	1.11(0.94–1.31)**	1.03 (0.89–1.20)	1.01(0.87–1.19)	1.01 (0.88–1.15)

* p <0.05

** p <0.20

CD = Chagas disease, ECG = Electrocardiogram, AMI = acute myocardial infarction, ACEI = Angiotensin converting enzyme inhibitor, ARA = angiotensin receptor antagonists, NT-ProBNP = N-terminus of natriuretic peptide prohormone of type B.

In the adjusted model (Table 5), the factors associated with a lower QoL per domain were: 1—Physical domain: increasing age (OR: 0.95; CI: 0.91–0.99) and using the medication class ACEI (OR: 0.89; CI: 0.80–0.99); 2—Psychological domain: no covariates remained in the final model; 3—Social Relations domain: a history of AMI (OR: 0.75; CI: 0.61–0.92) and being female (OR: 1.09; CI: 0.99–1.21) were associated with better QoL. Although with borderline significance (p = 0.057), the female gender was maintained in the final model considering their clinical and scientific relevance; 4—Environment domain: age increase was also associated with worse QoL (OR: 0.94; CI: 0.91–0.97) and use of the medication class ARA (OR: 1.15; CI: 1.04–1.26) associated with better scores. By the interpretation of the Odds Ratio (OR), in the inflated beta regression, all the factors that have OR < 1 were associated with a lower QoL, while values > 1 indicated a better QoL. Residual analyses indicated that the model assumptions were satisfied for the final regression models.

**Table 5:** Results of multivariate analysis using inflated beta regression factors associated with QoL by domain of the WHOQOL-BREF scale.

Variables	WHOQOL-BREF Domains							
	Physical		Psychological		Social Relations		Environment	
	OR	IC 95%	OR	IC 95%	OR	IC 95%	OR	IC 95%
Age (in 10 years)	0.95	0.91–0.99*	-		-		0.94	0.91–0.97*
Use of ACEI (Yes)	0.89	0.80–0.99*	-		-		-	
Gender (female)	-		-		1.09	0.99–1.21	-	
History of AMI	-		-		0.75	0.61–0.92*	-	
Use of ARA (Yes)	-		-		-		1.15	1.04–1.26*

*p<0.05

AMI = acute myocardial infarction, ECG = electrocardiogram, ACEI = angiotensin converting enzyme inhibitor, ARA = angiotensin receptor antagonists.

## Discussion

Patients with Chagas Disease (CD) from the SaMi-Trop cohort had a lower QoL in the Environment domain followed by the Physical domain, and a higher score in the Social Relations domain. The subjective perception of QoL was not associated with the severity of the disease using as a marker the presence of CCC in the evaluated population.

Other investigations that assessed QoL in CD using the same instrument (WHOQOL-Bref) found similar results with a lower score in the Environment domain and a higher score in the Social Relations domain [9,26,27]. The lower QoL score in the Environment domain may be an impact marker of the social determinants of health on the individual perception of QoL. These determinants accompany the entire historical process of CD transmission [28] and are aspects evaluated in the Environment domain as financial resources, available social and public health services, opportunities for participation in leisure and recreation activities, and are almost always neglected in remote regions. On the other hand, a higher QoL score was observed in the Social Relations domain, demonstrating a good perception of social relations, social support, and sexual activity. People who live with their partners have greater family and social support [29], and in the present study 63.7% of the participants are married or live with a partner. Another aspect is that in small towns, people tend to know each other and communicate more, thus providing greater capacity to face chronic health conditions. This involvement of patients with CD in collective activities such as social and friendship relationships may have a positive impact on personal relationships, which in turn may reflect the better perception of QoL in this domain [30].

The perception of QoL in patients with CD identified in this study does not differ from the general population. In a study with adult users enrolled in primary health care [31] and in a household survey conducted with the elderly, results similar to the findings of this study were also observed [32].

Although worse QoL in patients with CCC were expected to be identified, no statistically significant difference was observed between individuals with other cardiopathies and chronic chagasic cardiomyopathy, demonstrating that, in this investigation, cardiac impairment can impact QoL independent of the patient being chagasic or not. A review study [8] reported that having CD with cardiac manifestation is associated with worse QoL, a result also observed by Santos-Filho et al. [9] who used the same scale of this investigation (WHOQOL-Bref) to assess QoL. In this research, controversial results with the literature regarding the association of CCC with QOL may be related to the characteristics of the SaMi-Trop cohort, which includes all patients with chronic Chagas disease and all with some cardiac impairment. Thus, the comparison performed here included homogeneous individuals regarding cardiac impairment. Therefore, it is suggested that other studies evaluating QoL in patients with CD with established cardiomyopathy, and patients in the indeterminate form, be conducted.

In the present investigation it was observed that the increase in age is associated with a lower QoL score in the Physical domain, a result also found in other investigations [33–35]. It is known that elderly patients have physiological changes in the aging process with a higher prevalence of chronic conditions [36,37], which may impact on physical aspects such as mobility, daily life activity, and greater dependence on medication, being aspects assessed in the WHOQOL Physical domain. The increase in age also showed association with lower QoL in the Environment domain. Precarious socioeconomic conditions and residing in remote and rural areas can cause difficulties in accessing aspects included in the Environment domain such as transportation, financial resources, participation in reaction and leisure activities, as well as the availability and quality of health and social care. The lack of quality and availability of these resources in these remote regions may have influenced the perception of a lower QoL in the Environment domain associated with increasing age, considering that these resources are widely used in this age group. With aging there is an increase in the use of essential public services, such as health, mainly due to the increase in comorbidities and the use of medications [38].

In this investigation we found a high prevalence of self-related hypertension, above 60%, but a statistically significant difference was not found compared to those who answered the QoL evaluation interview and those who did not. In Brazil, a similar prevalence of hypertension in CD patients was observed by Santos-Filho et al, with 67% of hypertension [9]. However, prevalence data for the general population ranged from 15.9% to 31.2%, in both genders, this frequency increased with age and was more prevalent in less literate individuals [39].

Another factor associated with a lower quality of life in the Physical domain was the use of the ACEI class medication. Among patients who used ACEI, 93.6% had arterial hypertension and 32.8% had high NT-ProBNP levels suggestive of heart failure. Although ACEI is the medication of choice for heart failure in CD [11], it is known that adverse reactions may occur due to medication intolerance, which may have influenced a lower QoL in the Physical domain, considering the aspects evaluated in this dimension such as fatigue, low capacity for work, pain, and discomfort. According to the Brazilian guideline for CD [11], for the treatment of heart failure a combination of diuretics, ACEI, or ARA are used, with the latter being used in case of intolerance to ACEI.

In this sense, the patients of this investigation who used ARA had better QoL in the Environment domain. Both the ACEI and ARA medication classes are available in the Brazilian public health system, on the National Medication List (RENAME) [40], being offered by the Public Health System (SUS) and distributed by primary health care. However, although medications are available free of charge, there may be a shortage in the municipalities, causing the use of one medication class over another. This difficulty of access to the medication of first choice may impact on the pharmaceutical assistance of patients with CD, considering that most of them depend on medication distributed by the SUS. A review study [8] emphasizes the effectiveness of non-surgical conservative treatments, such as the adequate use of medications for cardiac manifestations, as determinants of QoL in patients with CD.

Patients with a history of acute myocardial infarction (AMI) presented lower QoL scores in the Social Relations domain. Studies have shown that the presence of comorbidities may also negatively influence QoL [41]. The post-infarction period may require changes in lifestyle and activities of daily living [42]. This can impact the personal relationships and sexual activity that are aspects evaluated in the Social Relations domain of the WHOQOL scale. During the occurrence of AMI and also in the recovery period there is strong social support, but this decreases over time [43] which may contribute to a lower perception of QoL in this domain. Another aspect is that the functional capacity of patients and the extent of cardiac involvement are also determinants of QoL in CD [8]. In the study population, 58.6% of the patients had electrocardiographic changes typical for CD, that is, they already had cardiomyopathy associated with the presence of another cardiac comorbidity (a history of AMI). This may have been a determining factor in the perception of a worse QoL in the Social Relations domain for this population.

Finally, it was observed that the women presented better QoL in the Social Relations domain. Although with borderline statistical significance, the gender variable was maintained in the final model due to its scientific relevance. A better female QoL was observed in studies involving patients with CD and other chronic diseases [35,44]. However, there are controversial results in that females were a predictor of worse QoL [7,9,26]. In the present study, a better perception of QoL in the Social Relations domain by women may be related to the fact that they have better social and interpersonal integration and also greater family involvement, which may favor their QoL [35], especially in this dimension.

This investigation presents important QoL indicators for health planning for patients with CD, but it is necessary to discuss some points. The first refers to the inclusion criteria of patients in the QoL interview. The eligibility criteria were the patient to be able to understand the QoL questions and conduct the self-assessment of their QoL. However, using this criteria, less than one third of individuals took part in the QoL evaluation. They were unable to complete the questionnaire because of the difficulty in understanding the instrument’s questions. In order to minimize this problem, differential bias comparing the individuals who had the QoL evaluated or not was evaluated. Individuals who had the QoL evaluated were different in some sociodemographic characteristics such as mixed skin color, older and more literacy, with more time since CD diagnosis, and more prevalent use of amiodarone, however no statistically significant differences were found in clinical outcomes. Thus, patients who did not have their QOL assessed could have worse QoL and worse clinical outcomes, not measured in this investigation. Caution should be exercised in extrapolating these results to other patients with CD, since this study fails to analyze the possibly of more severe patients or those out of the network of health services and with worse social determinants. A second limitation refers to the fact that there was no change in the class of medications due to adverse reactions. QoL may have been influenced by the use of medication that generated intolerance. Finally, the SaMi-Trop cohort consists of patients with established CC, which directly affects the perception of QoL and makes it difficult to extrapolate these results to groups with asymptomatic or indeterminate CD.

Even with some limitations, the findings of this study demonstrate useful information for the organization of health services and the care line for patients with CD, including the diagnostic and therapeutic component, as well as context factors, that may impact the care provided with consequent health disparities. In the organization and planning of services for this population it is important to be careful not to contribute to the health disparity, considering that worse health indicators are observed among socially disadvantaged people, in particular members of marginalized racial / ethnic groups and people economically disadvantaged. Usually, these are individuals who are in the margin of assistance and health assessments. Health equity should be our primary commitment, thus seeking to reduce or eliminate health disparities while being mindful of their social determinants. Ensuring health equity means giving special attention to the needs of those individuals at higher risk of health problems, based on social conditions [45]. It is expected that the findings of this study reinforce the importance of QoL indicators to improve planning and allocation of resources for better patient care, seeking improvements in the diagnosis and clinical management of patients with CD.
